# Intracranial Hemorrhage After Mechanical Thrombectomy: Proximal Versus Distal MCA M1 Occlusions

**DOI:** 10.1002/brb3.71562

**Published:** 2026-06-25

**Authors:** Dogan Hasan, Aytac Emrah, Balgetir Ferhat, Akpinar Cetin Kursad

**Affiliations:** ^1^ Interventional Neurology, Department of Neurology, Samsun City Hospital, Faculty of Medicine, Samsun University Samsun Turkey; ^2^ Interventional Neurology, Department of Neurology, Faculty of Medicine, Fırat University Firat University Elazığ Turkey

**Keywords:** mechanical thrombectomy, ischemic stroke, MCA M1 occlusion, intracranial hemorrhage

## Abstract

**Background and Purpose:**

Intracranial hemorrhage (ICH) is a recognized complication of mechanical thrombectomy that may affect outcomes. Whether the occlusion site within the MCA M1 segment (proximal vs. distal) influences the risk and subtype distribution of post‐procedural ICH remains uncertain. We compared the frequency and subtypes of ICH between proximal and distal MCA M1 occlusions and evaluated their impact on 90‐day clinical outcomes.

**Methods:**

We retrospectively analyzed consecutive patients from two stroke centers who underwent mechanical thrombectomy for isolated MCA M1 occlusion. Patients were classified as proximal or distal. Post‐procedural ICH was assessed on 24‐h non‐contrast CT (or earlier if NIHSS worsened by ≥ 4 points) and categorized as hemorrhagic infarction (HI, Types 1–2) or parenchymal hematoma (PH, Types 1–2). Ninety‐day outcomes were evaluated using the modified Rankin scale (mRS).

**Results:**

Among 178 patients (107 proximal, 71 distal), successful reperfusion (mTICI 2b–3) was achieved in 89.1%. HI was more frequent in proximal than distal occlusions (27.1% vs. 12.6%, *p* = 0.037), mainly due to HI1 (12.1% vs. 1.4%, *p* = 0.009). PH and symptomatic ICH rates were similar between groups. In logistic regression analysis, occlusion site was not an independent determinant of post‐thrombectomy hemorrhage (OR 1.52, 95% CI 0.80–2.91, *p* = 0.20). At 90 days, functional outcomes did not differ significantly between groups (mRS 0–2: 39.2% proximal vs. 47.9% distal, *p* = 0.271).

**Conclusion:**

Hemorrhagic infarction is more frequently observed in proximal MCA M1 occlusions, likely due to involvement of lenticulostriate arteries. However, this does not translate into a worse functional outcome.

## Graphical abstract

Hemorrhagic infarction was more common in proximal MCA M1 occlusions, mainly HI1, but this increased rate did not translate into worse 90‐day functional outcome following mechanical thrombectomy.

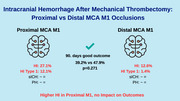

## 1 Introduction

Intracranial hemorrhage (ICH), both symptomatic and asymptomatic, is a frequent complication following mechanical thrombectomy (MT). In a meta‐analysis of five randomized clinical trials, the rate of symptomatic ICH (sICH) was 4.4% in the MT group and 4.3% in the control group (Goyal et al. 2016). In the DAWN and DEFUSE‐3 trials, symptomatic bleeding occurred in 6% (vs. 3% in control) and 7% (vs. 4% in control), respectively (Nogueira et al. 2018; Albers et al. 2018). Asymptomatic hemorrhage has been reported in approximately 20% of cases (Akpinar et al. 2021; Sengeze et al. 2022). Intracranial bleeding may develop due to iatrogenic vascular dissection or perforation during the procedure, or as a result of hemorrhagic transformation of the ischemic core (Goyal et al. 2016; Saver et al. 2016; Mehra et al. 2012). Advanced age (> 80 years), elevated NIHSS score (> 10), large ischemic lesion volume, hypertension and hyperglycemia, low ASPECTS score (< 6), intravenous (IV) or intra‐arterial (IA) fibrinolysis, and poor collateral circulation are factors that increase the risk of post‐thrombectomy hemorrhage (Flint et al. 2013; Christoforidis et al. 2009; P. Khatri et al. 2008; Soize et al. 2013).

Only a few studies have evaluated isolated proximal versus distal MCA M1 occlusions in terms of post‐thrombectomy hemorrhage (Ko 2021; Salahuddin et al. 2022; S. H. Kim et al. 2021). A recent study found no statistically significant difference in hemorrhagic transformation rates between proximal and distal occlusions of the MCA M1 (Ko 2021). Salahuddin et al. (2022) reported higher rates of hemorrhagic transformation in proximal M1 occlusions, along with lower rates of excellent clinical outcomes and a higher number of device passes. In the study by S. H. Kim et al. (2021), no significant difference was found between patients with proximal and distal MCA M1 occlusions in terms of hemorrhagic transformation. Despite these findings, the evidence comparing proximal and distal M1 occlusions and their association with clinical outcomes remains limited.

In the present study, we aimed to investigate the frequency of hemorrhagic transformation—both symptomatic and asymptomatic—and bleeding types (hemorrhagic infarction [HI]/parenchymal hematoma [PH]) in patients who underwent thrombectomy for isolated proximal and distal MCA M1 occlusions and to assess their effect on clinical outcomes.

## 2 Materials and Methods

### 2.1 Study Design and Patients

We retrospectively included patients who underwent MT for MCA M1 occlusion between January 2021 and January 2023 at two regional stroke centers. Both centers are staffed by two stroke neurologists who perform all interventional procedures, ensuring standardized treatment. Patients who received IV thrombolysis prior to MT were excluded to isolate the hemorrhagic effects attributable to the endovascular procedure. All patients achieving successful recanalization (mTICI 2b–3) were included. Additional exclusion criteria were tandem lesions, pretreatment CT Alberta Stroke Program Early CT Score (ASPECTS) < 6, or contraindications to endovascular therapy. The study was approved by the local ethics committee, and the requirement for written informed consent was waived due to its retrospective nature.

### 2.2 Patient Classification and Data Collection

MCA M1 occlusions were classified as proximal or distal. Proximal M1 occlusion was defined as an occlusion involving the origin of the lenticulostriate arteries (LSAs), whereas distal M1 occlusion referred to occlusions occurring beyond the lenticulostriate artery take‐off. Baseline demographics (age, sex), medical history (atrial fibrillation, hypertension, congestive heart failure, coronary artery disease, hypercholesterolemia, diabetes mellitus, smoking, and previous stroke), and procedural data (revascularization time, symptom‐onset to groin‐puncture time, mTICI score, and number of recanalization attempts) were collected from the Interventional Neurology Database. Neurological assessments at admission were performed by a stroke neurologist using the NIHSS. Early ischemic changes were evaluated on pretreatment CT using ASPECTS (Pexman et al. 2001).

### 2.3 Endovascular Therapy

Patients with MCA occlusion detected by CTA were considered for MT regardless of proximal or distal location. Arterial access was achieved via the right femoral artery. A combined technique (stent retriever + aspiration, Solumbra) was attempted in all patients; if not feasible, a direct aspiration first‐pass technique (ADAPT) was used. If recanalization was not achieved with one technique, the alternative method was applied. Stent retrievers, including Solitaire (Medtronic Neurovascular, Irvine, CA, USA), Thrombite (Zylox Tonbridge, Zhuhai, China), Trevo (Stryker Neurovascular, Fremont, CA, USA), and Aperio (Acandis, Pforzheim, Germany), were used, and device selection and transitions between techniques were left to the discretion of the interventional neurologists.

### 2.4 Imaging and Hemorrhage Assessment

All patients underwent an urgent non‐contrast CT and CTA at admission. A nonenhanced cranial CT scan was routinely repeated within 24 h after treatment (or earlier in cases of rapid neurological deterioration). When hemorrhage or contrast staining was suspected on the 24‐h CT, an additional follow‐up CT scan was obtained at 48–72 h to confirm the final hemorrhagic classification. Final hemorrhagic transformation (HI/PH) categorization was based on the combined assessment of these imaging studies.

sICH was defined as any intracranial bleeding associated with neurological deterioration, that is, an increase of ≥ 4 points in NIHSS. Cerebral hemorrhages were classified according to ECASS criteria: PH1, hematoma within the ischemic field involving < 30% of the infarcted area with mild mass effect; PH2, hematoma within the ischemic field involving > 30% with significant mass effect; and any intraparenchymal hemorrhage remote from the ischemic field. In addition, sICH was defined as PH1, PH2, subarachnoid hemorrhage (SAH), or intraventricular hemorrhage associated with NIHSS worsening > 4 within 24 h (Fiorelli et al. 1999).

Collateral status was graded according to Tan et al. (2009): 0, no collaterals; 1, filling of < 50% of the occluded MCA territory; 2, filling of 50%–100%; and 3, complete collateral filling of the occluded territory.

### 2.5 Statistical Analysis

After coding, the data were analyzed using SPSS (version 22.0, SPSS Inc., Chicago, IL, USA). Continuous variables were expressed as mean ± standard deviation (or median [interquartile range] as requested by the journal because they did not fit the normal distribution), and categorical data were expressed as numbers (%). In statistical analyses, the conformity of the measured variables to normal distribution was evaluated by the Kolmogorov–Smirnov test. Student's t‐test was used for the comparison of continuous variables conforming to normal distribution, and the Mann–Whitney *U* test was used for the comparison of continuous variables not conforming to normal distribution. Pearson's chi‐square test and Fisher's exact test were used to compare the data obtained by counting. A univariate logistic regression analysis was initially conducted. Variables significant in univariate analysis were entered into a multivariate logistic regression analysis using the backward likelihood ratio (LR) method. Statistical significance level was accepted as *p* < 0.05 for all tests.

## 3 Results

A total of 178 consecutive patients (78 males [43.8%], 100 females [56.2%]) who underwent MT for MCA M1 occlusion were included. The mean age was 69.7 ± 11.9 years (median 67, range 21–88), mean NIHSS score was 14.5 ± 4 (median 15, range 5–27), and mean ASPECTS score was 8.4 ± 1.2. Mean symptom‐onset‐to‐recanalization time was 324.1 ± 115.2 min. Overall, successful recanalization (mTICI 2b–3) was achieved in 89.1% of patients, and a good clinical outcome (mRS 0–2) at 90 days was achieved in 42.8% of patients. The rates of favorable outcomes did not differ significantly between proximal and distal M1 occlusions (39.2% vs. 47.9%, *p* = 0.271). There was no significant difference between the two groups regarding the collateral scoring system.

**FIGURE 1:**
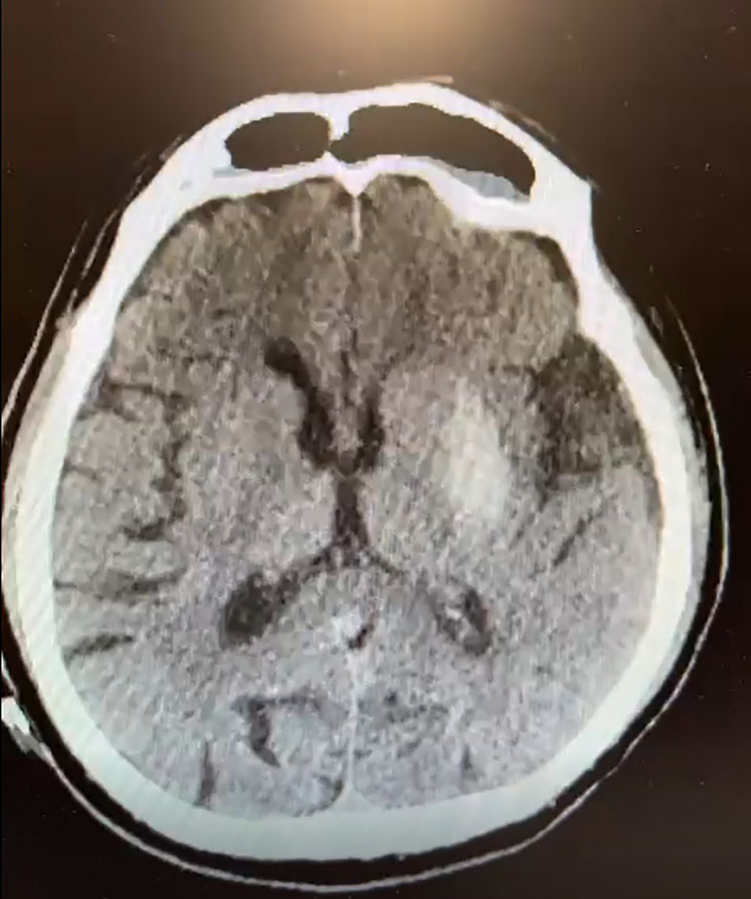
Type 2 hemorrhagic infarction in the brain, computed tomography scan taken after mechanical thrombectomy in a patient with proximal occlusion of MCA M1.

**FIGURE 2:**
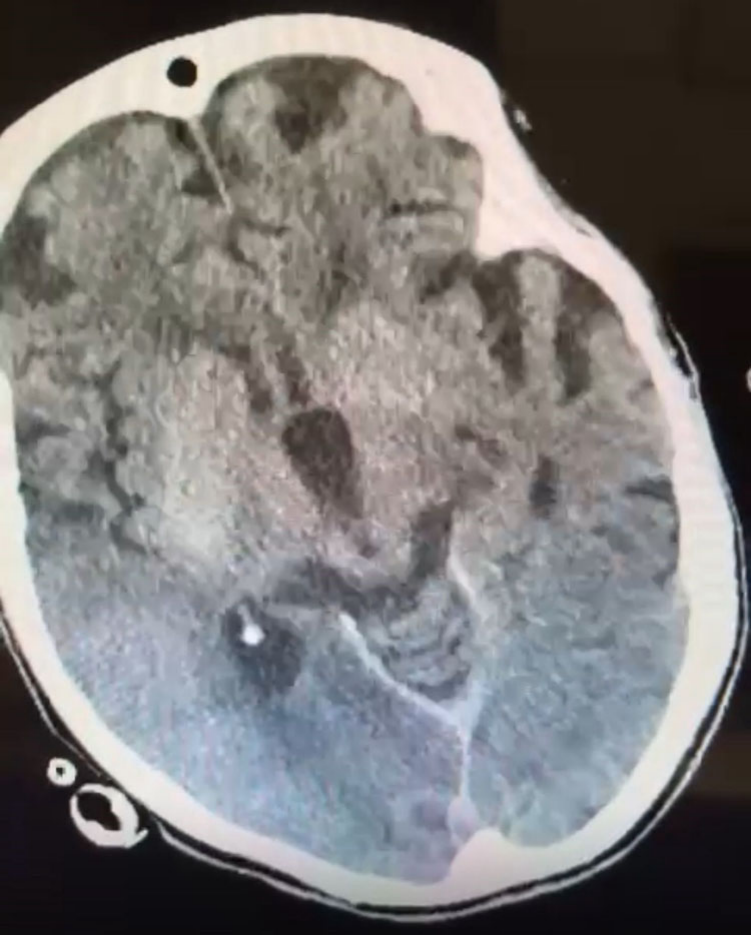
Type 2 hemorrhagic infarction in the brain, computed tomography scan taken after mechanical thrombectomy in a patient with distal occlusion of MCA M1.

Of the 178 patients, 107 had proximal MCA M1 occlusions and 71 had distal occlusions. Demographic, clinical, and angiographic characteristics of patients with proximal and distal MCA M1 occlusions are summarized in Table 1, and 24‐h brain CT findings are presented in Table 2 (Figures 1 and 2).

**TABLE 1:** Demographic and clinical characteristics and angiographic findings of MCA M1 proximal and distal occlusion.

	MCA M1 proximal (*n* = 107)	MCA M1 distal (*n* = 71)	*p*‐value
Side (right/left) (%)	57%/43%	43.7%/56.3%	0.081
Age	68.8 ± 12.3	71.1 ± 12.2	0.691
NIHSS	15.1 ± 3.5	13.7 ± 4.5	0.232
CT ASPECT	8.5 ± 1.2	8.4 ± 1.2	0.311
mRS (mean ± SD)	3.3 ± 1.8	2.9 ± 2	0.582
Good functional outcome (mRS 0–2), *n* (%)	42 (39.2%)	34 (47.9%)	0.271
No bleeding (%)	62.6%	71.8%	0.059
Symptom‐recanalization	319.04 ± 126.6	331.9 ± 95.8	0.323
Number of passes	1.3 ± 0.8	1.4 ± 0.8	0.460
mTICI 2b‐3 (%)	90.1%	88.1%	0.306
First pass (%)	44%	39%	0.632
Gender (M/F) (%)	46.7/53.3%	39.4/60.6%	0.996

Abbreviations: CT, computed tomography; F, female; M, male; MCA, middle cerebral artery; mRS, modified Rankin scale;

NIHSS, National Institutes of Health Stroke Scale.

**TABLE 2:** 24‐h brain tomography findings.

	MCA M1 proximal (*n* = 107)	MCA M1 distal (*n* = 71)	*p*‐value
No bleeding	67 (62.6%)	51 (71.8%)	0.059
HI total	29 (27.1%)	9 (12.6%)	**0.037**
HI Type 1	13 (12.1%)	1 (1.4%)	**0.009**
HI Type 2	16 (15%)	8 (11.2%)	0.512
PH total	7 (6.5%)	5 (7.0%)	0.911
PH Type 1	4 (3.7%)	3 (4.2%)	0.892
PH Type 2	3 (2.8%)	2 (2.8%)	0.991
SAH	4 (3.7%)	6 (8.4%)	0.211
sICH	3 (2.8%)	2 (2.8%)	0.996

Abbreviations: HI, hemorrhagic infarction; PH, parenchymal hematoma; SAH, Subarachnoid hemorrhage; sICH, symptomatic intracranial hemorrhage.

Bold values indicate statistically significant results (p < 0.05).

On follow‐up CT scans, no hemorrhage was observed in 62.6% of proximal cases and 71.8% of distal cases (*p* = 0.059). sICH occurred in three patients (2.8%) in the proximal group and two patients (2.8%) in the distal group (*p* = 0.996).

HI was significantly more frequent in proximal MCA M1 occlusions compared to distal occlusions (27.1% vs. 12.6%, *p* = 0.037). Subgroup analysis revealed that HI Type 1 was significantly higher in the proximal group (12.1% vs. 1.4%, *p* = 0.009), whereas HI Type 2 rates were not significantly different between groups (15.0% vs. 11.2%, *p* = 0.512).

PH rates were similar: PH1 (3.7% vs. 4.2%, *p* = 0.892) and PH2 (2.8% vs. 2.8%, *p* = 0.991). SAH rates were 3.7% in proximal and 8.4% in distal occlusions (*p* = 0.211).

According to the final model of the multivariate logistic regression analysis, the variables that had a statistically significant effect on hemorrhage were symptom‐to‐recanalization time (OR = 1.006, *p* < 0.001) (Table 3). The Omnibus Test of Model Coefficients indicated that the model was statistically significant overall (*p* < 0.001), and the Hosmer–Lemeshow Test showed that the model had a good fit to the observed data (*p* > 0.05).

**TABLE 3:** Logistic regression model of potential factors on hemorrhage.

	Univariate				Multivariate			
	Odds ratio	CI lower	CI upper	*p*	Odds ratio	CI lower	CI upper	*p*
Age	0.997	0.972	1.024	0.840				
Sex								
Female (ref)								
Male	1.615	0.863	3.021	0.134				
MCA								
MCA M1 distal (ref)								
MCA M1 proximal	1.522	0.796	2.912	0.204				
mRS	1.197	1.011	1.416	0.037				
NIHSS	1.068	0.983	1.159	0.118				
CT ASPECT	0.747	0.566	0.986	0.040				
Hyperdense artery								
No (ref)								
Yes	0.812	0.434	1.517	0.513				
Fazekas								
0 (ref)								
1	2.461	0.259	23.343	0.432				
2	1.714	0.178	16.424	0.640				
3	2.000	0.205	19.500	0.550				
4	2.666	0.157	45.141	0.496				
Symptom‐recanalization time	1.006	1.003	1.009	**< 0.001**	1.006	1.003	1.009	**< 0.001**
Hemisphere								
Right (ref)								
Left	0.905	0.486	1.687	0.754				
First pass								
No (ref)								
Yes	0.889	0.448	1.765	0.736				
Pass count	1.209	0.846	1.726	0.297				
mTICI								
2b (ref)								
2c	0.889	0.370	2.136	0.792				
3c	0.498	0.221	1.120	0.092				

Abbreviations: CT, computed tomography; MCA, middle cerebral artery; mRS, modified Rankin scale; NIHSS, National Institutes of Health Stroke Scale.

Bold values indicate variables retained in the final multivariate logistic regression model.

## 4 Discussion

In the present study of patients with MCA M1 occlusion undergoing MT, no significant differences were observed between proximal and distal M1 subgroups regarding first‐pass effect, successful recanalization (mTICI 2b–3), distal embolization, sICH, or favorable 90‐day outcome. However, HI was significantly more frequent in proximal occlusions (*p* = 0.037), primarily driven by HI1, while PH rates were comparable. sICH occurred in only a few patients, which makes it difficult to draw firm conclusions about differences between the groups. Multivariate analyses further demonstrated that occlusion site was not an independent determinant of post‐thrombectomy hemorrhage. These findings suggest that the anatomical distribution of LSAs may shape bleeding patterns after reperfusion without translating into worse functional outcomes when timely and effective recanalization is achieved.

In studies focusing on anterior circulation strokes, MCA M1 is reported as the most frequent vascular occlusion (mean 69%) (Badhiwala et al. 2015). In studies including both anterior and posterior circulation strokes, the frequency of MCA M1 occlusion is approximately 49.7% (Hansen et al. 2015). LSAs arising from the M1 segment of the MCA do not communicate with other perforators and are therefore considered “end arteries.” They are subdivided into medial LSAs, which originate anterior to the cerebral artery, and lateral LSAs, which arise lateral to the MCA. Lateral LSAs supply the posterolateral head of the caudate nucleus, anterior part of the body of the caudate nucleus, caudate tail, anterior putamen, and lateral globus pallidus. In addition, the globus pallidus, the lower part of the genu, and posterior limb of the internal capsule, and the posteromedial lentiform nucleus are supplied by perforators from the anterior choroidal artery, Heubner's recurrent artery, and the posterior communicating artery (Decavel et al. 2012). As LSAs are end arteries without collateral circulation, infarction rapidly develops in the deep subcortical region (basal ganglia) after flow interruption, and progression can be fast. Following recanalization, reperfusion into these ischemic territories predisposes them to hemorrhagic transformation (Loh et al. 2009).

Hemorrhagic transformation after MT is known to be a complex, multifactorial process. Na^+^/K^+^‐ATPase activity ceases within minutes after ischemic stroke due to energy failure, resulting in breakdown of the blood–brain barrier through cellular and metabolic pathways (Rossi et al. 2007). The prolonged ischemic period and the subsequent inflammatory response further aggravate blood–brain barrier disruption (R. Khatri et al. 2012). Consequently, the loss of autoregulation leads to extravasation of blood into reperfused ischemic regions (Raychev et al. 2015). Therefore, the timing of reperfusion emerges as a decisive factor in determining whether ischemia‐related injury progresses to hemorrhage. Early reperfusion may limit ischemic injury to vascular structures and reduce the risk of reperfusion bleeding, whereas late reperfusion increases the risk of hemorrhage by reintroducing blood into infarcted tissue (Teal and Pessin 1992)

Large randomized controlled trials support this association. In EXTEND‐IA (0%), SWIFT PRIME (0%), and ESCAPE (3.6%), procedure duration was under one hour, while in REVASCAT, where the mean procedure time exceeded one hour, the sICH rate was higher (4.9%) (Goyal et al. 2016; Nogueira et al. 2018; Albers et al. 2018; Akpinar et al. 2021). In another study, every 10‐minute delay in recanalization increased the risk of intracerebral hemorrhage in the basal ganglia by approximately 11%. In the same study, hemorrhage restricted to the basal ganglia was associated with successful recanalization (Raychev et al. 2015).

During thrombectomy, iatrogenic factors such as mechanical pressure on the vessel wall, endoluminal trauma, or vessel perforation may also lead to intracerebral hemorrhage (Teal and Pessin 1992). Preclinical and clinical studies have demonstrated that newer‐generation devices, particularly the Solitaire, cause less endothelial injury and are associated with lower rates of hemorrhage compared with earlier devices like Merci (Mehra et al. 2012; Liu et al. 2016). Reported rates of post‐thrombectomy hemorrhage vary, with asymptomatic hemorrhage around 16%, symptomatic hemorrhage about 7%–8%, and vascular perforation up to 5% (Daou et al. 2015; Yoon et al. 2013). Prospective series have described hemorrhagic transformation within the ischemic area in up to 40% of patients (Hornig et al. 1986; Okada et al. 1989), and histopathological analyses suggest even higher rates at the microscopic level, up to 65% (Moulin et al. 1994).

Other studies have identified specific predictors of hemorrhage. One study reported ICH in 10% of patients and symptomatic bleeding in 2%. Risk factors for symptomatic hemorrhage included > 3 device passes, baseline ASPECTS ≤ 7, and symptom‐onset‐to‐puncture time exceeding 280 min (Lee et al. 2023). In another series of 89 patients undergoing MT, hemorrhagic transformation was seen in 42 patients (47.2%). Asymptomatic bleeding occurred in 31 patients (HI1 11.2%, HI2 14.6%, PH1 4.5%, PH2 4.5%), while symptomatic bleeding occurred in 11 patients (PH2 7.8%, IVH 4.5%) (Jiang et al. 2015). Similarly, in a recent study, sICH was only 2% in both the MT and IVT+MT groups. In the IVT‐only group, HI1, HI2, PH1, and PH2 occurred at rates of 12%, 11%, 5%, and 4%, respectively; in the IVT+MT group, these rates were 10%, 13%, 6%, and 7% (Bracard et al. 2016). Differences among these studies likely reflect variations in eligibility criteria, study design, baseline stroke severity, symptom‐to‐recanalization times, and demographic characteristics (Sussman and Connolly 2013).

Regarding isolated MCA occlusions, findings are heterogeneous. Some studies found no significant difference in clinical outcomes between proximal and distal MCA M1 occlusions (Ko 2021; S. H. Kim et al. 2021; Tian et al. 2019; Y. S. Kim et al. 2019), whereas others suggested worse outcomes in proximal occlusions (Behme et al. 2015; Y. S. Kim et al. 2019). Data on overall hemorrhagic complications after thrombectomy remain limited. In one study, no significant difference was found between proximal and distal MCA M1 occlusions regarding hemorrhagic transformation (S. H. Kim et al. 2021). Conversely, another study reported significantly higher hemorrhagic transformation in proximal compared to distal occlusions (37.4% vs. 23.5%, *p* = 0.02). Among 261 patients, 35% had proximal and 65% distal occlusions. Proximal occlusions were associated with lower rates of excellent clinical outcomes (24.7% vs. 39.5%), higher number of passes (2.4 ± 1.4 vs. 2.1 ± 1.4; *p* = 0.02), and higher HR score (18 [S. H. Kim et al. 2021; Pexman et al. 2001; Fiorelli et al. 1999; Tan et al. 2009; Badhiwala et al. 2015; Hansen et al. 2015; Decavel et al. 2012; Loh et al. 2009; Rossi et al. 2007) vs. 16 [Ko 2021; Salahuddin et al. 2022; S. H. Kim et al. 2021; Pexman et al. 2001; Fiorelli et al. 1999; Tan et al. 2009; Badhiwala et al. 2015; Hansen et al. 2015; Decavel et al. 2012]; *p* = 0.003) (Salahuddin et al. 2022). Loh et al. (2009) also reported increased risk of PH (OR 6.7, 95% CI 1.02–183.3) in thrombectomy patients with basal ganglia involvement. Taken together, these heterogeneous results emphasize that hemorrhagic complications after thrombectomy are influenced by multiple interacting factors, including symptom‐to‐recanalization time, baseline NIHSS, baseline ASPECTS, number of passes, blood glucose levels, and comorbidities.

Given the heterogeneous findings reported in previous studies on proximal versus distal M1 occlusions, our study adds clarity by examining hemorrhagic transformation subtypes with angiographically confirmed occlusion‐site definitions. Unlike much of the earlier literature that primarily emphasized functional outcomes or technical aspects of the procedure, our analysis focuses directly on the distribution of HI and PH subtypes. In addition, because our cohort consisted exclusively of MT‐only patients, the hemorrhagic patterns presented here more specifically reflect the effects of the endovascular procedure itself, without the confounding influence of prior IV thrombolysis.

Since the lenticulostriate artery territory is frequently involved, intra‐infarct hemorrhage in this region is expected following vessel recanalization. Importantly, such hemorrhages often occur within the infarcted tissue and usually do not cause additional neurological deterioration. In our study, HI Type 1 was observed more frequently in proximal M1 occlusions; however, this did not correspond to worse functional outcomes. Nevertheless, the number of HI Type 1 events in our cohort was limited, and this should be taken into account when interpreting this subgroup finding. The ECASS I trial similarly reported that the presence of HI was not associated with poor functional prognosis (Fiorelli et al. 1999). Previous studies also suggest that petechial hemorrhages without mass effect are usually clinically benign (Hornig et al. 1986; Okada et al. 1989; Moulin et al. 1994). In contrast, PH (particularly PH2) has been more strongly linked to poor neurological outcomes in prior literature (Fiorelli et al. 1999; Sussman and Connolly 2013). Taken together, these findings support the conclusion that higher HI rates in the proximal group did not adversely affect clinical outcomes in our cohort. Nonetheless, such hemorrhages may be relevant in situations involving thrombolysis or acute stenting in tandem lesions. In addition, a hyperdense artery sign or hyperdense middle cerebral artery sign (HMCAS) is known to be associated with clot burden and has been reported to be more common in proximal M1 occlusions, potentially affecting recanalization difficulty and bleeding risk (Merlino et al. 2023). In our study, HMCAS was included in the regression model evaluating predictors of hemorrhage; however, it was not found to be associated with post‐thrombectomy hemorrhage.

We excluded patients who received IV thrombolysis in order to isolate hemorrhagic complications directly attributable to MT. Although this limits the generalizability of our findings to real‐world mixed cohorts, recent evidence suggests that IV thrombolysis does not substantially alter the risk or pattern of hemorrhagic transformation in M1 occlusions. In the study by Ryu et al. (2024), neither the overall rate of hemorrhagic transformation nor the distribution of hemorrhage subtypes (HI‐1, HI‐2, PH‐1, PH‐2) differed significantly between MT‐only and IVT+MT groups, and even symptomatic hemorrhage did not increase with IVT. Therefore, while our cohort represents MT‐only patients, the hemorrhagic patterns observed here are broadly consistent with those reported in studies that included patients treated with bridging therapy.

The main limitations of this study include the small sample size, the retrospective two‐center design, and the lack of evaluation of etiological differences. Although all eligible patients treated during the study period were included, the retrospective nature of the work makes it difficult to completely rule out selection bias and potential unmeasured confounding. In addition, the low number of hemorrhage events in certain subgroups may render these results statistically more fragile and prone to Type I error. Similarly, the very low number of sICH cases limits statistical power, and the nonsignificant result should not be interpreted as equivalence. For this reason, we believe that the subgroup findings should be interpreted with appropriate caution. Although patients who received IV thrombolysis were excluded to allow a clearer evaluation of hemorrhagic events specifically related to MT, this approach may reduce the generalizability of the findings, as many real‐world M1 occlusion patients undergo bridging therapy. Device selection and procedural technique variability may also act as potential confounders, as differences in thrombectomy technique, device type, or number of passes could influence the risk of hemorrhagic transformation after reperfusion.

## 5 Conclusion

HI occurred more frequently in proximal MCA M1 occlusions than in distal occlusions; however, this did not translate into differences in 90‐day functional outcomes, nor was occlusion site an independent predictor of hemorrhage. These findings suggest that anatomical factors, such as lenticulostriate artery involvement, may shape bleeding patterns, but their clinical impact appears limited when timely and effective recanalization is achieved. As this was a retrospective observational study with a limited number of hemorrhagic events, the results should be interpreted cautiously and confirmed in larger prospective studies.

## Author Contributions

**Dogan Hasan**: conceptualization, investigation, writing – original draft, visualization, validation, methodology, software, formal analysis, project administration, data curation, resources, writing – review and editing. **Aytac Emrah**: supervision, software, writing – review and editing, data curation, formal analysis, writing – original draft. **Balgetir Ferhat**: software, data curation, resources, formal analysis, writing – review and editing, writing – original draft. **Akpinar Cetin Kursad**: conceptualization, writing – original draft, data curation, resources, supervision, writing – review and editing, visualization, methodology, validation.

## Funding

The authors have nothing to report.

## Ethics Statement

This study was approved by the Samsun University Non‐Interventional Clinical Research (GOKA‐EK) ethics committee.

## Conflicts of Interest

The authors declare no conflicts of interest.

## Data Availability

The data that support the findings of this study are available from the corresponding author upon reasonable request.
